# Supplementary material for: Musashi-1 and miR-147 Precursor Interaction Mediates Synergistic Oncogenicity Induced by Co-Infection of Two Avian Retroviruses
Source: Cells. 2022 Oct 21;11(20):3312. doi: 10.3390/cells11203312 (PMC9600308; doi:10.3390/cells11203312)
Supplement: Supplementary file 1 [file cells-11-03312-s001.zip › cells-1969996-supplementary.pdf]

Table S1. Primer sequences used in qPCR analysis.

Gene	Primer sequences	Product (bp)
KIAA1199	F: GTCTCCATCCACCACACCTTCT<br>R: ACAAGCAGTCCAAGGCAGTGA	161
NF-κB p50	F: AAGCAGGCAGAGGTGGTAGAA<br>R: TTGTCATCTCCTTCAGCAGCAG	110
NF-κB p65	F: ACCACCACCACAACCACAATGC<br>R: AGCGGCGTCGATGGTATCAA	114
EGFR	F: TCAGTCGCCAGAAGGAGTGT<br>R: CCAGAGCAGGTTGTGTTGTATG	142
MSI1	F: TTTGTGGGTGGTCTGTCAGTGA<br>R: CGGTGTCGGTTGGTTGTCTTG	116
Pri-miR-147	F: ACTGAGAGCGAATTGAATACCG<br>R: AGGCAACAGAAACTTGTCACTT	152
GAPDH	F: GAACATCATCCCAGCGTCCA<br>R: CGGCAGGTCAGGTCAACAAC	132
U6	F: CTCGCTTCGGCAGCACA<br>R: AACGCTTCACGAATTTGCGT	94

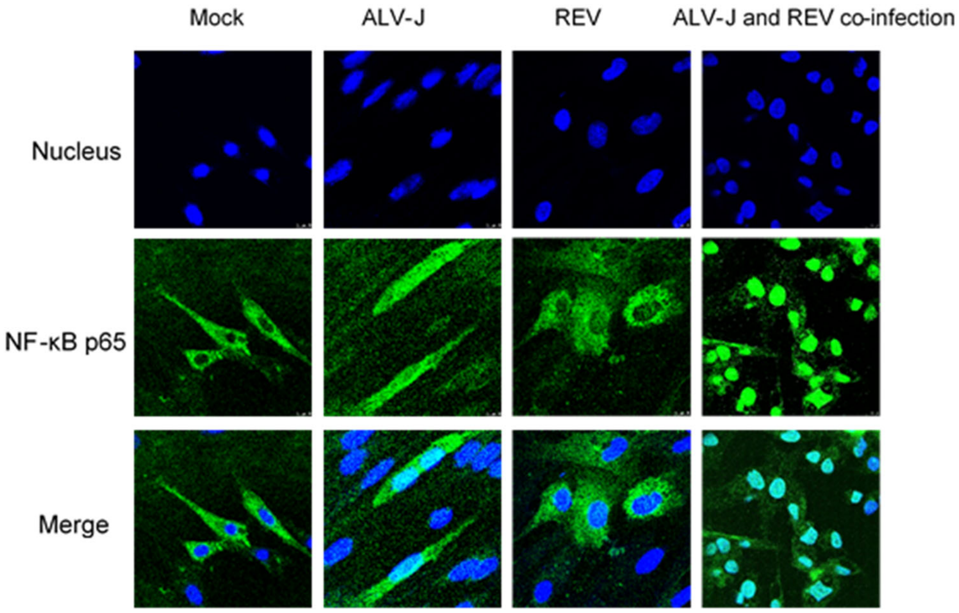

Figure S1. Compared with single infection, translocation of NF-κB to the nucleus was significantly observed in CEF co-infected ALV-J and REV at 72 hpi.
